# Clinical comparison of three SARS-CoV-2 nucleic acid amplification tests for routine diagnostic testing

**DOI:** 10.1016/j.heliyon.2023.e22112

**Published:** 2023-11-07

**Authors:** Tetiana Garmatiuk, Christine Gränitz-Trisko, Charlotte Sochor-Geischläger, Theresa Polsterer, Francesca Caselotto, Lukas Willitsch, Birgit Reinhardt, Wolfgang Huf

**Affiliations:** a Department of Laboratory Medicine, Hietzing Hospital, Wolkersbergenstraße 1, 1130, Wien, Austria; b Abbott GmbH, Max-Planck-Ring 2, 65205, Wiesbaden, Germany; c Karl Landsteiner Institute for Clinical Risk Management, Wolkersbergenstraße 1, 1130, Wien, Austria

**Keywords:** SARS-CoV-2, Real-time PCR, Alinity m, Respiratory viruses

## Abstract

**Background:**

Cycle threshold (Ct) values from SARS-CoV-2 nucleic acid amplification tests have been used to estimate viral load for treatment decisions. Additionally, there is a need for high-throughput testing, consolidating a variety of assays on one random-access analyzer.

**Objectives:**

In this study, the clinical performance of the Alinity m SARS-CoV-2, RealTi*m*e SARS-CoV-2, and GeneXpert Xpress SARS-CoV-2/Flu/RSV assays was assessed.

**Methods:**

Alinity precision and detection rates were evaluated using a dilution series of the Alinity m SARS-CoV-2 positive control. In a retrospective study, 7 remnant external quality assessment (EQA) specimens and 200 remnant nasopharyngeal swab specimens (100 positive and 100 negative) were tested in the three assays.

**Results:**

Alinity had 100 % detection rate at 50 copies/mL and high reproducibility (Ct value coefficient of variation ≤3.1 %). All three assays correctly detected positive and negative EQA samples with comparable Ct values (max difference 2.38) and high linearity. In patient samples, positive percent agreement was 95 % (95 % CI 89–98 %) and negative percent agreement was 100 % (95 % CI 96–100 %) for Alinity, compared to the other two assays. Four specimens detected on Alinity m but not RealTi*m*e or Xpert had Ct values above 40. Assay results were highly correlated (r ≥ 0.94). Ct values (after addition of 10 unread cycles to the reported Ct of RealTi*m*e) were comparable across the three assays.

**Conclusions:**

Alinity m had high precision and accuracy and Ct values comparable to those of the RealTi*m*e and Xpert assays. The assays could be used interchangeably, with no need for adjustment of patient management decisions based on Ct values from each assay.

## Highlights

• 100 % detection rate at 50 copies/mL with Alinity m SARS-CoV-2.
• Alinity m SARS-CoV-2 showed high reproducibility with CV% of Ct values ≤ 3.1 %.
• Percent agreement between Alinity m, RealTime and Xpert assays was 95–100 %.
• Results suggest slightly higher sensitivity of Alinity m versus RealTi*m*e and Xpert.
• Low mean Ct differences of 1.10–2.55 between Alinity m, RealTime, and Xpert.

## 1 Introduction

During the COVID-19 pandemic, the cycle threshold (Ct) value of SARS-CoV-2 nucleic acid amplification tests (NAATs) gained prominence as a way to semi-quantify viral load and guide patient management, despite continuing controversy regarding its utility [[1], [2], [3], [4], [5]]. A Ct value of 30 has been used to make clinical decisions on the duration of patient quarantine or discharge from hospital in absence of clinical symptoms [6]; however, intra-assay variability between multiple target gene regions of the test methods, combined with inter-assay variability, present challenges for the clinical interpretation of Ct values and their application to decision making [[7], [8], [9]].

We evaluated the Ct values and clinical performance of the recently launched Abbott Alinity m SARS-CoV-2 assay compared to two SARS-CoV-2 RT-PCR assays currently used in our laboratory for routine testing of all patients admitted to the hospital (Abbott RealTi*m*e SARS-CoV-2) or for urgent sample testing (Cepheid GeneXpert Xpress SARS-CoV-2/Flu/RSV).

## 2 Methods

### 2.1 Study design

This retrospective study was performed in the Department of Laboratory Medicine, Hietzing Hospital, Vienna, Austria. All clinical samples were deidentified prior to use in the study.

### 2.2 NAAT assays

The Alinity m SARS-CoV-2 assay (Abbott Molecular Inc., Des Plaines, IL, USA; “Alinity”) is indicated for the qualitative detection of SARS-CoV-2 in patients with or without signs and symptoms of COVID-19 infection. The assay can be run with nasopharyngeal, oropharyngeal, or nasal swabs, including pooled samples and saliva specimens [10]. Alinity is run on the Alinity m system, a fully automated, random-access analyzer with a time to first result of less than 2 h that reports approximately 1000 results in 24 h. The RealTi*m*e SARS-CoV-2 assay (Abbott Molecular Inc.; “RealTime”) is run on the m2000 batch analyzer with a turnaround time of approximately 6 h and is indicated for the qualitative detection of SARS-CoV-2 in nasopharyngeal and oropharyngeal swabs from patients suspected of COVID-19 infection [11]. The GeneXpert Xpress SARS-CoV-2/Flu/RSV assay (Cepheid, Sunnyvale CA, USA; “Xpert”), run on the GeneXpert system with a low throughput and a turnaround time of approximately 1 h, and is indicated for the qualitative detection and differentiation of SARS-CoV-2, influenza A, influenza B, and respiratory syncytial virus (RSV) viral RNA in nasopharyngeal swabs, nasal swabs, or nasal wash/aspirate specimens from patients suspected of respiratory viral infection [12]. All assays were performed according to the manufacturers’ instructions.

### 2.3 Analytical performance assessment

To assess Alinity precision and detection rates around the limit of detection (LOD) at 100 copies/mL [10], a dilution panel was prepared using the Alinity m SARS-CoV-2 positive control diluted in 0.9 % NaCl solution to concentrations of 400, 300, 200, 100, and 50 copies/mL. Each panel member was tested in 20 replicates; the 100 copies/mL dilution was tested in 40 replicates. Assay precision was assessed by calculating means, standard deviations (SD), and coefficients of variation (CV) of Ct values. Detection rates were also calculated.

Seven leftover external quality assessment (EQA) specimens from the Austrian Association for Quality Assurance and Standardization of Medical and Diagnostic Tests (OEQUASTA), the national EQA provider in Austria, were tested retrospectively. The EQA panel comprised two diluted SARS-CoV-2 positive swab specimens (including one alpha variant), one SARS-CoV-2 positive viral culture supernatant sample, one negative control (0.9 % NaCl solution), and one negative diluted swab specimen containing human RNA. The viral culture supernatant sample was tested undiluted and diluted (1:10 and 1:100) [13]. The leftover specimens had been stored at −80 °C for 6 months prior to testing on the three assays.

### 2.4 Clinical comparison of the three assays

A total of 200 remnant nasopharyngeal swab specimens collected over the previous 4 months and stored at −25 °C, with valid Ct values from one of the three assays, were analyzed with the other two assays. The samples were collected during monitoring of hospitalized patients and screening of newly admitted patients. The patient cohort included patients with respiratory symptoms as well as patients without respiratory symptoms that were admitted to the hospital for other reasons. For the analysis, 100 negative specimens and 100 positive specimens (Ct values 16.90–40.99) were randomly selected based on the Alinity results. As all remnant clinical specimens were anonymized prior to the study and no patient-specific information was collected, approval by an ethics committee was deemed unnecessary.

### 2.5 Data and statistical analysis

As Ct values are non-standardized and none of the assays could be considered the reference method, we used Pearson correlation to compare the analytical and clinical performance of the three assays. Statistical evaluation was conducted by Microsoft Excel and the add-in software Analyse-it.

## 3 Results

### 3.1 Analytical performance of alinity

The observed detection rates for all concentrations of the dilution panel were ≥97.5 % (Table 1). Additionally, Alinity had high reproducibility, with a Ct value CV of ≤3.1 %. In testing leftover EQA specimens, all positive and negative samples were correctly identified by all three assays (Table 2). Comparable Ct values were observed across the three assays with a maximum difference of 2.38. Linearity was high for the three dilution series samples, with Pearson's correlation coefficients ranging between −0.999 and −1.000 (data not shown).

**Table 1:** Detection Rates with the Alinity m SARS-CoV-2 Assay.

Target concentration (copies/mL)	Replicates tested (n)	Replicates detected (n)	Percentage of replicates detected	Mean Ct ± SD	Ct CV%
400	20	20	100 %	34.20 ± 1.06	3.1 %
300	20	20	100 %	34.12 ± 0.75	2.2 %
200	20	20	100 %	34.91 ± 0.89	2.6 %
100	40	39	97.5 %	36.13 ± 0.87	2.4 %
50	20	20	100 %	37.78 ± 1.00	2.7 %

Ct: cycle threshold; CV: coefficient of variation; SD: standard deviation.

**Table 2:** Evaluation of leftover samples of the austrian association for quality assurance and standardization of medical and diagnostic tests (OEQUASTA) [13].

EQA Specimen	Dilution Series	Viral Load (copies/mL)	Expected Result	Alinity Result (Ct value)	RealTi*m*e^a^ Result (Ct value)	Xpert Result (Ct value)
Human RNA	–	–	neg	neg	neg	neg
SARS-CoV-2	–	∼252,000	pos	25.52	26.51	27.90
SARS-CoV-2	1:10	∼22,300	pos	31.34	31.70	32.50
SARS-CoV-2 (alpha)	–	∼345,300	pos	26.76	28.35	28.90
0.9 % NaCl	–	–	neg	neg	neg	neg
SARS-CoV-2	1:1	∼96,100	pos	28.95	29.41	30.50
SARS-CoV-2	1:100	∼640	pos	36.00	36.73	38.30

EQA: external quality assessment; pos: positive result; neg: negative result.

a 10 unread cycles included.

### 3.2 Clinical performance of alinity against comparators

In testing 200 clinical samples, Alinity had a positive percent agreement (PPA) of 95 % (95 % CI 89–98 %) and a negative percent agreement (NPA) of 100 % (95 % CI 96–100 %; Table 3) compared to RealTi*m*e and Xpert. Of the 100 positive specimens, 94 yielded positive results with all three assays. Of the remaining 6 specimens, 4 samples with Alinity Ct values above 40 were negative on RealTi*m*e and Xpert. Two Alinity positive specimens with Ct values of 39.04 and 40.76 were found to be positive with either one of the other comparator assays. All 100 negative samples with Alinity were also negative with RealTi*m*e and Xpert.

**Table 3:** Agreement between alinity, RealTi*m*e, and xpert assays.

	Alinity + RealTi*m*e + Xpert +	Alinity + RealTi*m*e + Xpert -	Alinity + RealTi*m*e - Xpert +	Alinity + RealTi*m*e - Xpert -	Alinity - RealTi*m*e - Xpert -		Alinity/RealTi*m*e Agreement	Alinity/Xpert Agreement	RealTi*m*e/Xpert Agreement
Alinity + ^(n = 100)^	94	1^a^	1^b^	4^c^	–	PPA	95 % (CI 89–98 %)	95 % (CI 89–98 %)	99 % (CI 94–100 %)
Alinity - ^(n = 100)^	–	–	–	–	100	NPA	100 % (CI 96–100 %)	100 % (CI 96–100 %)	99 % (CI 95–100 %)

PPA: Positive percent agreement; NPA: Negative percent agreement; CI: 95 % confidence interval.

a Ct (Alinity): 39.04.

b Ct (Alinity): 40.76.

c Ct (Alinity): 40.41–40.99.

**Fig. 1:**
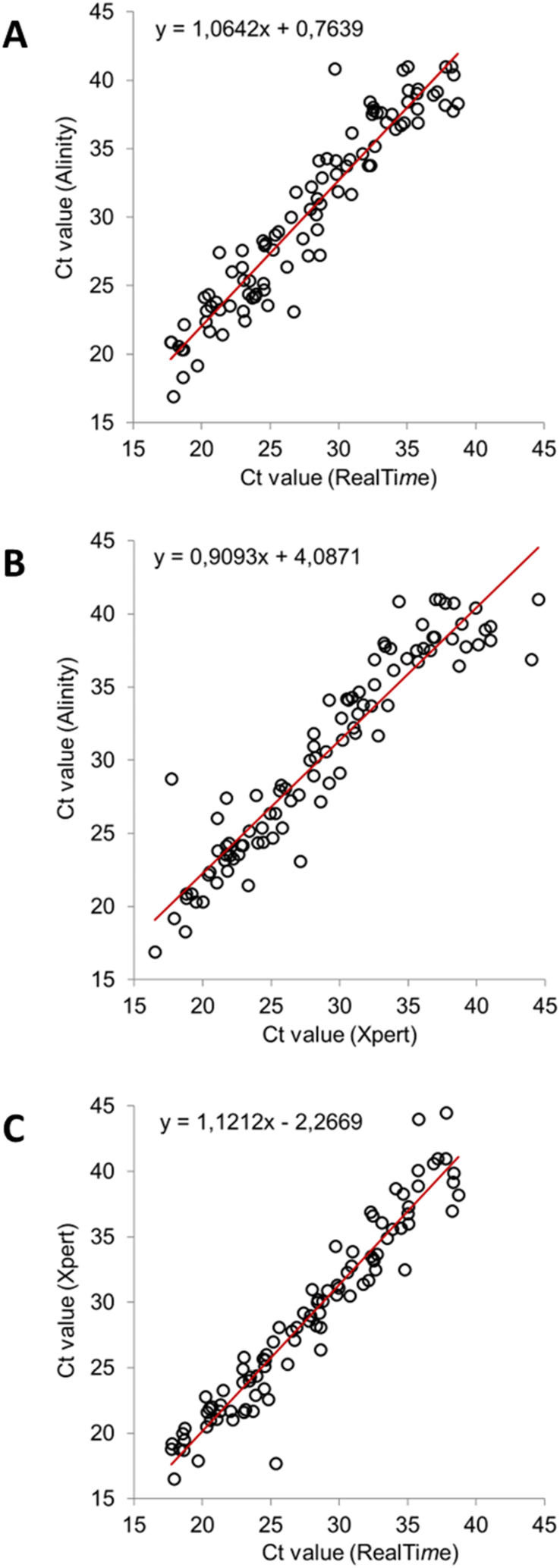
Correlation of Ct values for (A) Alinity and RealTi*m*e, (B) Alinity and Xpert, and (C) Xpert and RealTi*m*e. Ct values for RealTi*m*e include the 10 unread cycles.

The three assays showed high correlation (p < 0.0001), with r values of 0.95 between Alinity and RealTi*m*e, 0.94 between Alinity and Xpert, and 0.96 between RealTi*m*e and Xpert (Fig. 1A–C). With addition of the 10 unread cycles to the reported RealTime Ct values, Bland-Altman evaluations showed low mean Ct differences between the assays: 2.55 (Alinity – RealTi*m*e), 1.46 (Alinity – Xpert), and 1.10 (Xpert – RealTi*m*e). Individual specimen Ct values had a mean difference of 1.7 ± 0.1 (SE).

## 4 Discussion

We found that Alinity had a high precision with a Ct value CV of ≤3.1 %. This observation was similar to previous studies that reported Ct value CVs of ≤1.9 % [14,15]. In our study, the detection rate of 100 % at 50 copies/mL by Alinity exceeded the sensitivity claimed by the manufacturer. Our finding was concordant with other assessments of the LOD of Alinity using similar dilution panels and observing LODs of 50–100 copies/mL [[14], [15], [16]].

When testing leftover Austrian OEQUASTA EQA specimens including the SARS-CoV-2 variant alpha (B.1.1.7), we observed high accuracy of all three assays, matching the expected results. Ct values (after addition of the 10 unread cycles to the reported Ct of RealTi*m*e) were comparable across the three assays, with a maximum Ct difference of 2.38 across assays and samples, and showed high linearity. Of note, across all participants in the EQA with a variety of platforms, the reported differences between the highest and the lowest Ct values within the same samples ranged between 12.7 and 17.8, and some laboratories did not report linearity in the dilution series [13]. Our results confirmed the accurate detection of multiple SARS-CoV-2 variant lineages, including B.1.1.7, B.1.351, and P.1 variants, by Alinity and RealTi*m*e as demonstrated previously by testing dilution series of heat-inactivated virus cultures down to concentration levels of 0.73–2.85 log copies/mL. In that study, all dilutions across the different variants were reliably detected by both assays [17].

In our performance assessment, 100 samples were tested negative with all three assays resulting in 100 % negative percent agreement and suggesting high specificity for the assays evaluated in the study. This is important to correctly identify individuals without SARS-CoV-2 infection [18].

Significant and high correlation was seen across all three assays in our study, with correlation coefficients ≥0.94. Notably, the three assays produced similar Ct values, with mean Ct differences ranging between 1.10 and 2.55. This is of specific interest because in a recent study, the range of obtained Ct values across all evaluated test methods was approximately 30 Ct values per sample, respectively. In that study, two reference samples of about 6 and 7 Log copies/mL had been tested in 305 laboratories using overall 1109 test methods. Thus, for optimal interpretation of positive test results, it was recommended to anchor Ct values of individual test systems to reference materials of known viral RNA concentrations [9]. Similar to our results, a high correlation between Ct values of Alinity and RealTi*m*e (R^2^ = 0.95) and a mean difference of 4.14 Ct values were previously observed if including the unread cycles for RealTi*m*e [16]. Another study compared Alinity with other fully automated SARS-CoV-2 assays, Roche cobas® SARS-CoV-2 (cobas® 6800) and GeneXpert Xpress SARS-CoV-2, as well as with two semi-automated SARS-CoV-2 assays by Primerdesign (genesig®) and R-Biopharm (RIDA®GENE), respectively. The Ct values of the four assays correlated well with Alinity (r ≥ 0.946) while the CVs of the Ct values ranged from 0.7 % to 3.2 % for the fully automated assays compared to 5.8–6.6 % for the semi-automated tests, suggesting a higher precision of the fully automated assays. The median Ct values had a difference from Alinity of >7.6 Ct for the semi-automated assays compared to a difference of 3.1 Ct for cobas and 5.1 Ct for GeneXpert [19].

The results of our study suggest a slightly higher sensitivity of Alinity compared to RealTi*m*e and Xpert. Similarly, a comparison of Alinity, RealTi*m*e, and Seegene Allplex SARS-CoV-2 showed 100 % NPA and 100 % PPA while one of the two targets of an inhouse assay appeared to be slightly less sensitive [14]. A higher sensitivity was also observed for Alinity compared to Roche cobas SARS-CoV-2 (cobas 6800) as 37 out of 998 samples positive by Alinity were not detected by cobas 6800 while all 961 samples positive by cobas 6800 were also positive with Alinity [20]. Moreover, when testing a dilution series of a SARS-CoV-2 positive cell culture supernatant, Alinity was one of the two assays with the highest sensitivity among multiple commercial SARS-CoV-2 assays tested [21]. Finally, high concordance was also observed when comparing Xpert to Alinity m Resp-4-Plex (detecting SARS-CoV-2, influenza A/B, RSV) and Roche cobas Liat SARS-CoV-2/influenza A/B. Alinity m Resp-4-Plex exhibited the lowest LOD for SARS-CoV-2 at 26 copies/mL compared to 58 copies/mL for cobas Liat and 83 copies/mL for Xpert [22]. As many laboratories use multiple test methods for SARS-CoV-2 detection, it is important to understand how individual assays compare to each other.

Some authors suggested that results with high Ct values are potentially false positive results. This assumption is based on the observation that follow-up samples collected within 72 h or longer could confirm initial Alinity m results >37 Ct only in 13/40 (32.5 %) of cases and initial in-house assay results with 34–40 Ct only in 11/339 (3.2 %) of cases. However, as the median time interval between the index test and a positive follow-up test result was less than 1 day (22 h), the positivity rate of the follow-up sample may have been impacted by the interval length between the collection dates of the two samples [23]. Clinical significance of SARS-CoV-2 results with high Ct values should be interpreted in the context of the clinical history of a patient. Detection of viral RNA does not necessarily represent transmissible virus, and the relationship between timing of SARS-CoV-2 detection and duration of infectivity is not fully understood. SARS-CoV-2 detection is considered critically important in the early phase of infection when patients are highly infectious and need to be quarantined. In contrast, prolonged virus detection beyond day 10 after symptom onset or day of positivity [24] is likely to be of minor clinical relevance with a low risk of transmission expected [25].

Limitations of this study were that the detection rates were evaluated before the 1st WHO International Standard for SARS-CoV-2 RNA was available. Thus, the use of the Alinity m SARS-CoV-2 positive control to prepare the dilution panel may limit the ability to compare our findings to other SARS-CoV-2 assays. Furthermore, additional panel members with lower concentrations would have provided a better estimation of the actual LOD in this study. However, in previous studies, detection rates for viral RNA less than 50 copies/mL dropped below 95 % [14,15]. Samples with positive results on Alinity but negative on one or both of the two comparator assays could not be re-run due to limited sample volume.

## 5 Conclusions

Our study demonstrated that results from Alinity, run on the random-access Alinity m analyzer, correlated well with results from RealTi*m*e and Xpert in detecting SARS-CoV-2, providing similar Ct values. Thus, it can be used interchangeably as part of the clinical routine.

## Credit author statement

Tetiana Garmatiuk; Wolfgang Huf, Dr: Conceived and designed the experiments; Analyzed and interpreted the data; Wrote the paper. Christine Gränitz-Trisko, Dr.: Contributed reagents, materials, analysis tools or data. Charlotte Sochor-Geischläger; Theresa Polsterer; Lukas Willitsch: Performed the experiments.Francesca Caselotto: Conceived and designed the experiments. Birgit Reinhardt, Dr.: Analyzed and interpreted the data; Wrote the paper.

## Data availability statement

Data will be made available on request.

## Additional information

No additional information is available for this paper.

## Declaration of competing interest

The authors declare the following financial interests/personal relationships which may be considered as potential competing interests:Tetiana Garmatiuk reports article publishing charges and writing assistance were provided by Abbott Laboratories.
